# Ciguatera in the Indian Ocean with Special Insights on the Arabian Sea and Adjacent Gulf and Seas: A Review

**DOI:** 10.3390/toxins13080525

**Published:** 2021-07-27

**Authors:** Nazima Habibi, Saif Uddin, Marie-Yasmine Dechraoui Bottein, Mohd Faizuddin

**Affiliations:** 1 Environment and Life Sciences Research Center, Kuwait Institute for Scientific Research, Safat 13109, Kuwait; nhabibi@kisr.edu.kw; 2 Université Côte d’Azur, CNRS, ECOSEAS, UMR7035, Parc Valrose, 06108 Nice, France; y.bottein@gmail.com; 3 Gulf Geoinformation Solutions, Sharjah, United Arab Emirates; faiz_ud_din@yahoo.com

**Keywords:** the Indian Ocean, Arabian sea, Kuwait bay, Aden Gulf, Red Sea, Gulf of Aqaba, Andaman Sea, Bay of Bengal, seafood safety, foodborne disease

## Abstract

The dinoflagellates of the genus *Gambierdiscus* are found in almost all oceans and seas between the coordinates 35° N and 35° S. *Gambierdiscus* and *Fukuyoa* are producers of ciguatoxins (CTXs), which are known to cause foodborne disease associated with contaminated seafood. The occurrence and effects of CTXs are well described in the Pacific and the Caribbean. However, historically, their properties and presence have been poorly documented in the Indian Ocean (including the Bay of Bengal, Andaman Sea, and the Gulf). A higher occurrence of these microorganisms will proportionately increase the likelihood of CTXs entering the food chain, posing a severe threat to human seafood consumers. Therefore, comprehensive research strategies are critically important for developing effective monitoring and risk assessments of this emerging threat in the Indian Ocean. This review presents the available literature on ciguatera occurrence in the region and its adjacent marginal waters: aiming to identify the data gaps and vectors.

## 1. Introduction

Ciguatera poisoning (CP) is a syndrome caused by ingestion of coral reef fish and shellfish of tropical and subtropical regions, which has caused global concern. Some dinoflagellate species of the genera *Gambierdiscus* and *Fukuyoa* are known to produce ciguatoxins (CTXs); the organisms that consume these toxic algae accumulate CTXs that are transferred and biotransformed along the marine and human food chain. These lipid-soluble and heat-resistant toxins cause gastrointestinal, cardiovascular, and neurological disorders among humans consuming the CTX-contaminated seafood [1,2,3,4,5]. According to a study published in 2008, CP disease estimations are uncertain and often misdiagnosed [6]. However, few reports predict 2–10 million people are annually affected by CP [7,8].

Ciguatera poisoning is regarded globally as the most significant non-bacterial poisoning associated with fish consumption. It is usually limited to the consumption of toxic fish from regions between the latitudes 35° N and 35° S [9,10,11,12,13,14,15,16,17]. Studies have shown a strong positive correlation between *Gambierdiscus* abundance and algal macrophytes [18]. Some earlier studies proposed a standardized methodology for estimating *Gambierdiscus* abundance based on sampling macroalgae [19,20,21]. More recent studies brought to light the significant biases in *Gambierdiscus* cell distribution within the macrophytes. A 33–150% variation was reported among replicates [22,23,24]. Despite these limitations, some first-order estimates on *Gambierdiscus* distributions for large geographic regions have been attempted using the average abundances [11,25]. These have shown a global distribution spreading across oceans with 85% of *Gambierdiscus* density estimates <1000 cells g^−1^ wet weight algae, with <10% occurrence with 10–10,000 cells g^−1^ wet weight algae and <5% incidences where the concentration was >100,000 cells g^−1^ wet weight algae. With the growing global demands for seafood and international trade ease, CP concerns the population beyond the endemic regions and is increasingly becoming a global issue. Moreover, a greater threat is posed as there are no current antidotes for CP [26].

Historically, poisoning associated with seafood consumption was reported in different parts of the globe. It was first recounted in the West Indies as early as 1511 [27] and in the Gulf of Guinea in 1521, killing the Captains of the Spanish army [28]. It was also reported in the islands of the Indian Ocean in 1601 and various archipelagos of the Pacific Ocean in 1606 [27]. Additionally, in 1786, the surgeon of HMS Endeavour, en route to Australia, New Zealand, and the Pacific Islands, reported that the ship’s Captain was poisoned by ciguatoxins [29]. A year later, the ingestion of a local gastropod (*Livonia* sp.) was discovered to induce neurological symptoms in the Antilles of the Caribbean Sea [30]. In 1948, the organism *Gambierdiscus* (originally referred to as *Goniodoma* sp.) was described for the first time in Cabo Verde [31]. The term “ciguatera poisoning” was coined in Cuba (Caribbean Sea) after the ingestion of a marine snail (*Turbo pica*) locally known as cigua [32]. The historical facts suggested the tropical and subtropical Pacific and Indian Ocean insular regions, the tropical Caribbean region, and the continental reefs to be endemic to ciguatera [27].

In recent years, there has been an increase in the frequency of reports of toxic and harmful benthic algal blooms, predominantly *Gambierdiscus* with the presence of *Fukuyoa* and *Ostreopsis* blooms, throughout the world [33,34]. The interest in *Gambierdiscus* blooms has been heightened because of increased awareness of the effects of CP on human health. A growth in species abundance has already been observed in subtropical and temperate regions with the threat of global warming expected to further exacerbate the situation [35,36,37,38]. However, a recent article by Hallegraeff et al. [39] suggested that intensified monitoring efforts and heightened aquaculture activities are responsible for these perceived increases in HABs events that are not underpinned to be expanded as an empirical assumption. Ciguatoxins are reported globally, being described from new sites in the Canary Islands, Indian Ocean, Japan, and Western Gulf of Mexico [8,40,41,42,43,44,45]. Until recently, the records of *Gambierdiscus* in the Indian Ocean were scarce and restricted to the western tropical region, whereas now its presence has been found in the Northern part of the ocean [42]. There is paucity of information on ciguatera phenomena, including the occurrence of human poisonings, of toxins in seafood, and of the causative organisms, in the Indian Ocean in general, and the northern part in particular. In the present review, we gathered the published information on reported occurrences of *Gambierdiscus* and identified the research gaps related to its monitoring as a tool to manage this emerging hazard.

## 2. Results

### 2.1. Environmental and Global Pressures in the Northern Part of the Indian Ocean

The Indian Ocean is the third largest ocean enclosing densely populated landmasses: In the North, India, Bangladesh, Burma, Thailand, Pakistan, Iran, and Oman among others. It also includes regional seas in the North such as the Arabian Sea and the adjacent Lakshadweep Sea, Aden Gulf in the Red Sea, the Gulf of Aqaba and Suez, the Bay of Bengal and the Andaman Sea, as well as the Gulf of Oman and the Persian Gulf further north. Countries from the Indian Ocean region have experienced unusual climatological conditions such as cyclones, El Niño-Southern Oscillation events, and coral reef bleaching during the past two decades [46,47,48]. Benthic microalgae are particularly influenced by these disturbances especially the coral mortality, which provides a good substrate for the formation of algal turfs and associated epiphytes [49]. The study conducted by Quod et al. [46] reported a significant increase in the CP causative organisms in comparison to those reported in 1980 [47], following the coral bleaching event in 1998, thus posing an incremental risk of HABs in the region and drawing detrimental consequences to the marine biodiversity and human health [50].

The unfavorable effects of increasing atmospheric levels of carbon dioxide (CO_2_) and other greenhouse gases in the Indian Ocean and its marginal seas are leading towards acidification of the marine environment [48,51,52,53]. Supporting evidence for increased CO_2_ sequestration was drawn from increased marine primary productivity over the past decade [51,54]. The eutrophication of Kuwait bay and the nearby water bodies due to upwelling events is considered to be a predominant factor influencing the onset of algal blooms due to enriched nutrient conditions [54]. Other potential factors that have influenced the onset of blooms consist of coral bleaching, unusual variances in temperature, and calm conditions. Additional factors such as dust storms that carry micronutrients, domestic and industrial inputs, natural meteorological and oceanographic forcings, and the introduction of invasive species from ballast water discharge may all play a major role in the onset and expansion of HABs [41,45,55,56,57,58].

### 2.2. Ciguatera Causative Organisms Occurrence

**Figure 1:**
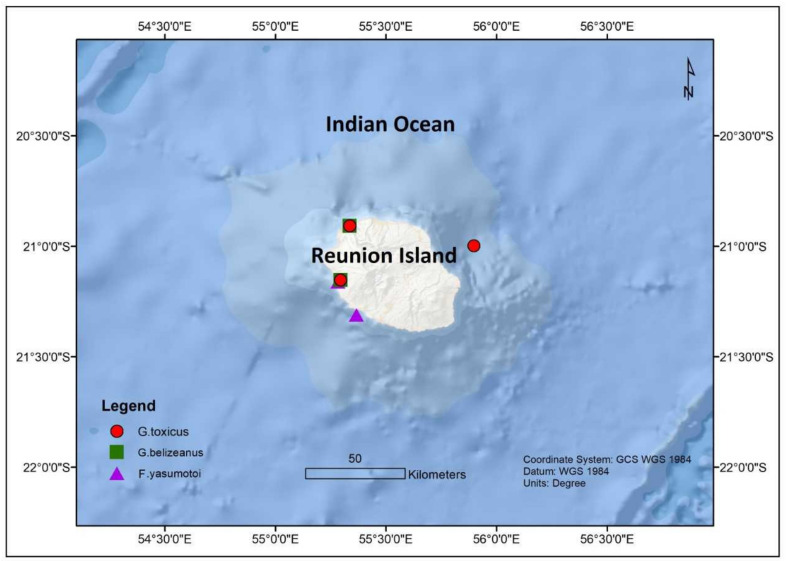
Spatial distribution of *Gambierdiscus* and *Fukuyoa* sp. from Reunion Island.

**Figure 2:**
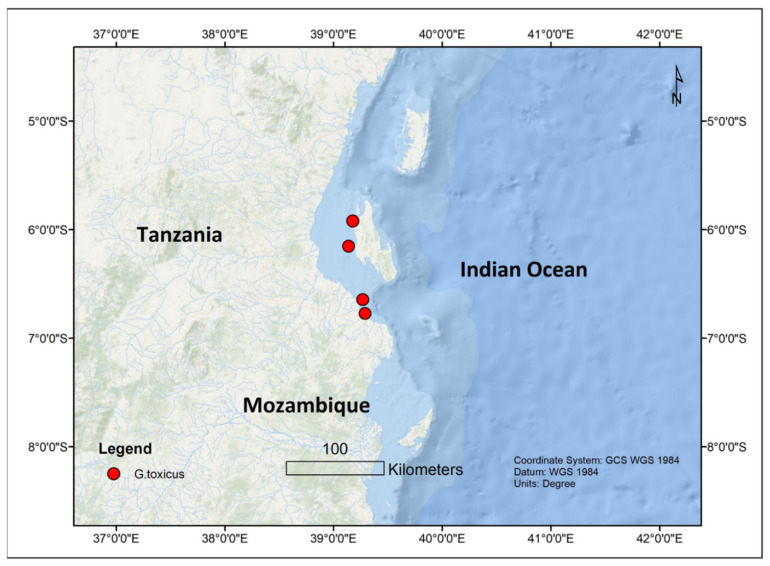
The occurrence of *Gambierdiscus* sp. from Tanzanian coastal area.

**Figure 3:**
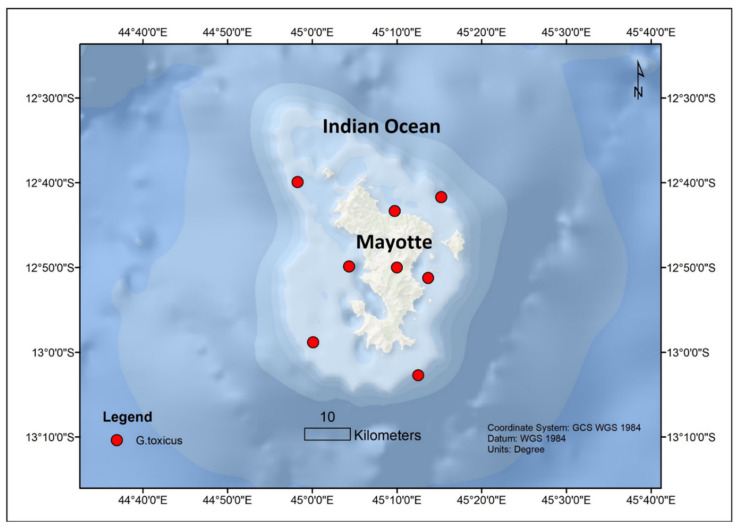
*Gambierdiscus* sp. reported from Mayotte Island, Indian Ocean.

The dinoflagellates *Gambierdiscus* and *Fukuyoa* are the causative organisms of CP worldwide [5]. The genus *Gambierdiscus* has a pantropic distribution with about 18 known species while *Fukuyoa* has 3 known species. Within the Indian Ocean, *Gambierdiscus* are more dominant in the western part. Of the several known *Gambierdiscus* sp., initially only *Gambierdiscus toxicus* was reported in Mayotte since *G. toxicus* was the first species that was described [50,59,60,61]. Later it was reported in La Reunion and Mauritius [62,63,64,65] as well (Figure 1). *Gambierdiscus toxicus* was also found in Mbudya Island, Oysterbay, Bawe Island, and Makoba Islands in Tanzania at depths of 5–10 m and temperatures ranging from 25 to 32 °C [66] (Figure 2). Thereafter, *Gambierdiscus yasumotoi* (now *Fukuyoa yasumotoi* (M.J. Holmes) [67]) and *Gambierdiscus belizeanus* were confirmed to occur in Mayotte in the Comoros archipelago [50] (Figure 3).

**Figure 4:**
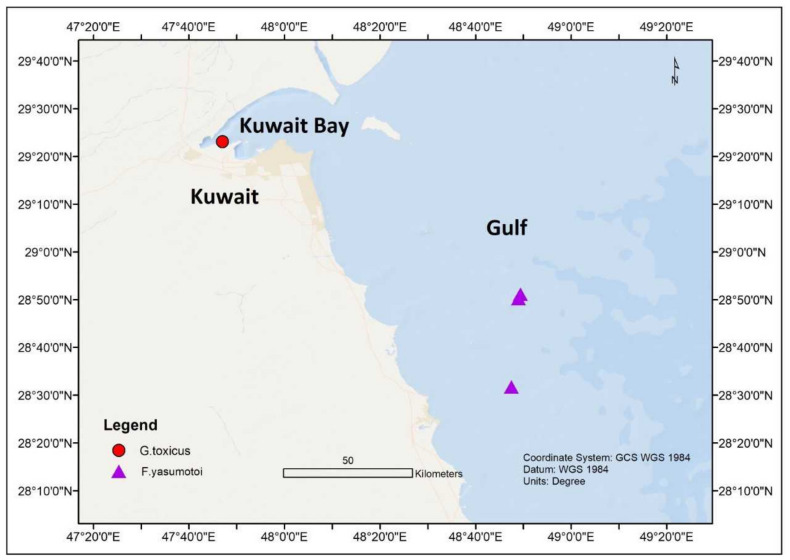
*Gambierdiscus toxicus* and *Fukuyoa yasumotoi* occurances in Kuwait Coastal waters.

In the northern part of the Indian Ocean, *Gambierdiscus* species have been noticed in the marginal seas of the Indian Ocean, such as the Arabian Sea [41,42,45,68]; Red Sea [41,42,43,44,45]; Gulf of Aqaba [42]; and Manora Channel, Pakistan [69]. An assessment carried out in Kuwait (Persian Gulf) from November 2012 to March 2013 showed the presence of *G. yasumotoi* in the shallow lagoons of Qit’at Julai’ah and southern coastal waters of Kuwait at 1–3 m depth [68,70] (Figure 4). The average seawater temperatures and salinity during this time were between 23.5–25 °C and 41.2–42.4 ppt, respectively. In another study, *G. toxicus* was also identified in Kuwait’s territorial water [41,45] (Figure 4).

**Figure 5:**
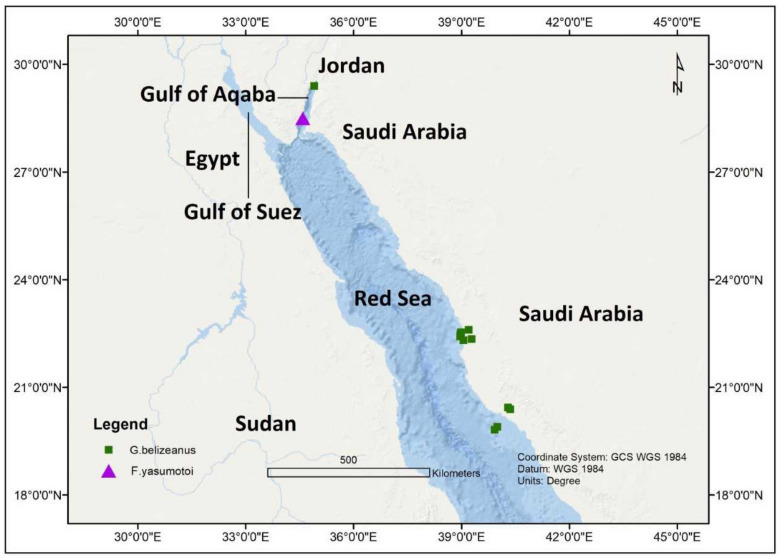
The Spatial distribution of *Gambierdiscus belizeanus* and *Fukuyoa yasumotoi* occurances in Red Sea and adjacent areas.

In an investigation from the Gulf of Aqaba, Jordan during October 2010, with a seawater temperature range of 24–25 °C and salinity of 40 ppt, *G. belizeanus* was reported from a depth of 1.5–2.0 m (Figure 5). The toxigenic *G. belizeanus* was reported from the Red Sea, Saudi Arabian coast during February 2012 and May 2013 [43,44] at a sampling depth between 0.4–0.8 m, with seawater temperatures and salinity range of 28.9–38.4 °C and 36.1–38.4 ppt respectively. The oxygen saturation during this period was very variable between 1.73–32.1%.

**Figure 6:**
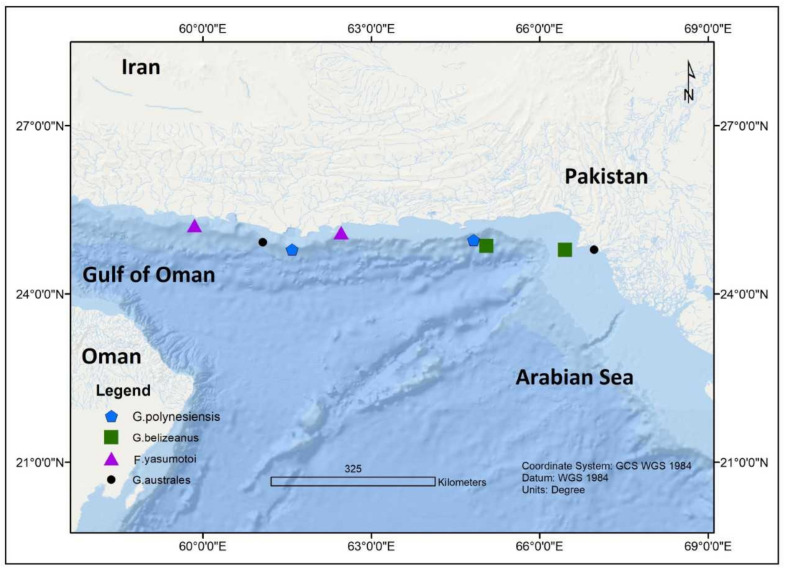
Occurrences of *Gambierdiscus* in Gulf of Oman and Pakistan’s coastal regions.

**Figure 7:**
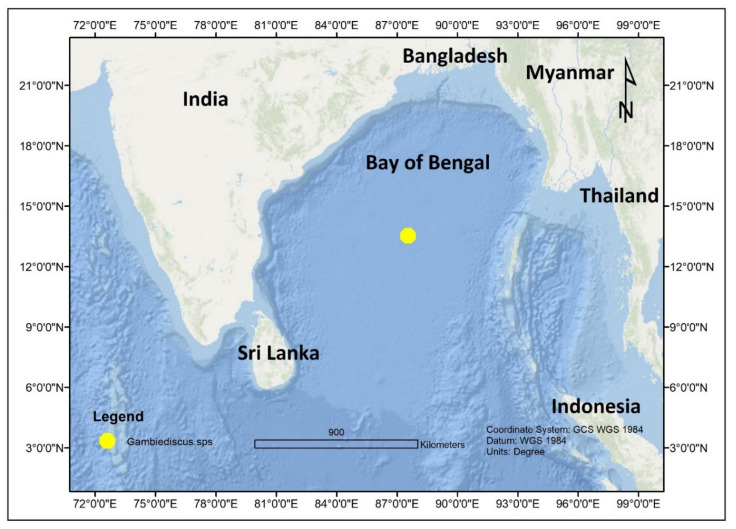
*Gambierdiscus* sp. reported from the Bay of Bengal, Indian Ocean.

In 2011, four *Gambierdiscus* species including *G. toxicus*, *G. belizeanus*, *G. polynesiensis*, *G. australes*, and *Fukuyoa yasumotoi* were also reported from the coastal waters of Pakistan in the northern Indian Ocean [69] (Figure 6). An unidentified *Gambierdiscus* sp. was also discovered in middle of the Bay of Bengal [71] (Figure 7). In Mangalore at the southwest coast of India, CP was first described during June 2015 outbreak involving red snapper(*Lutjanus bohar*) [72,73], and a second outbreak during September 2016 involving red snapper again [74]. In another incidence, CP was also reported from Trivandrum, India [75].

## 3. Discussion

### 3.1. Toxin Production by Ciguatera in Indian Ocean Region and Adjoining Marginal Seas

Ciguatoxins form a large group of toxins of 40 confirmed or suspected chemical analogs. They are classified into four types, according to both their geographical origin and chemical structure: The Pacific CTXs (P-CTX) of the type CTX3C and CTX4A, the Caribbean CTXs (C-CTX type), and the Indian CTXs (I-CTX type) [8,27]. The existence of the I-CTX type is still speculative and may be similar to that of the C-CTX. Their structural characteristic has not yet been elucidated. Six derivatives I-CTX 1–6 were identified with a molecular weight in Dalton of 1140.6, 1156.6, 1138.6, and 1154.6, however, their chemical structure is still unknown. Four of them were retrieved from a highly toxic *Lutjanus sebae* (Red Emperor) from the coast of the Republic of Mauritius through optimized gradient reversed-phase high-performance liquid chromatography-electrospray ionization mass spectrometry (LC/MS) methods, in combination with a radioligand receptor binding assay (r-RBA) [76,77], and two from in a bull shark (*Carcharhinus leucas*) implied in a fatal intoxication in Madagascar [76,77,78]. The lipid-soluble extracts retrieved from the edible fishes were reported to possess CTX activity and induced lethal symptoms in mice [77] and responded to the cell bioassay [78].

The toxicity of these *Gambierdiscus* species collected in the Pacific and/or the Caribbean was found to vary by over 2 orders of magnitude [11,79,80]. It remains to be confirmed that the same ranges are observed in the Indian ocean where only a few species have been analyzed for their toxin contents. Using in vitro cytotoxicity cell-based assays (CBA with neuroblastoma with N2a cells), the toxicity of two isolates of *G. belizeanus* from the Red Sea was estimated at 6.50 × 10^−5^ pg P-CTX-1 eq. cell^−1^ for RS2-B6 and 1.02 × 10^−5^ pg P-CTX-1 eq. cell^−1^ for RS3-B8. Toxin production was slightly higher in *G. belizeanus* strains from Tahiti (0.0246 fg P-CTX-3C eq. cell^−1^) [4,43,44]. As compared to *G. belizeanus,* the species of *G. polynesiensis*—0.017–4.4 pg P-CTX-3C eq. cell^−1^; *G. toxicus*—0.028 pg P-CTX-3C eq. cell^−1^ [4]; and *G. australes*—0.04 pg CTX-1 eq cell^−1^ [40] are more toxigenic.

More recently, Gambieric acid D was identified for the first time in the flesh of a bull shark (*Carcharhinus leucas*) employing the technique of liquid chromatography coupled with high-resolution mass spectrometry (HRMS). The toxicity was confirmed through mouse bioassays (Lowest dose = 72 mg equiv. stomach per mouse of 20–22 g) and neuro-2a cell-based assays (flesh—0.06 µg P-CTX-1 equiv./kg; stomach—92.78 µg P-CTX-1 equiv./kg; fins 1—0.12 µg P-CTX-1 equiv./kg; and fin 3—µg P-CTX-1 equiv./kg) [78]. Ciguatoxins were identified as CTX1B and 2,3-dihydroxy CTX3C; 51-hydroxy CTX3C; or positive by cell assay in Snapper fish (Lutjanidae) caught in the Indian Ocean and involved in 5 CP outbreak [81]. Fish samples from Sri Lanka were analyzed by the European Union Reference Laboratory for Marine Biotoxins (EURLMB) in Vigo, Spain, and the putatively contaminated fish samples were analyzed by liquid chromatography-tandem mass spectrometry (LC-MS/MS) according to a method published by [82] with slight modifications. The only available analytical standard was P-CTX-1B. Seven out of 11 samples tested positive for P-CTX-1B. In addition, other putative CTX variants were detected across most samples, however, they could not be confirmed as CTXs due to the lack of reference compounds. There are incidences of ciguatera poisoning due to the import of contaminated fish from the Indian ocean region, mainly India and Sri Lanka.

It is important to note that the investigations conducted so far have been limited in spatial coverage. Additional research is, therefore, required at a broader spatial scale to make meaningful conclusions regarding the occurrence, abundance, and temporal variability of ciguatoxins in the region.

### 3.2. Ciguatera Causative Organism’s Abundance

Globally, ciguatera causative organisms’ densities have been found to vary from few cells to thousands of cells per gr of substrate. *Gambierdiscus* is known as a slow-growing species compared to many other dinoflagellates; it takes about 5 months to increase its abundance. Chinain et al., [4] found a mean growth rate in *G. polynesiensis* as 0.13 ± 0.03 div d^−1^ at exponential phase in batch culture condition [4]. In the field, a 17 months lag time is estimated between a major environmental event (such as hurricane) and before it blooms and releases toxins into the environment and gets into the food chain [83]. During October 1998, a remarkably higher concentration of *G. toxicus* equivalent to 60,463 cells per gram of algae was recorded in Mayotte Islands (Comoros, southwest Indian Ocean) following a bloom event. This density is the highest ever recorded in the region and also the highest globally [50]. In a non-bloom event, the cell densities of *Gambierdiscus* were far less and often non-detectable.

In the western Indian Ocean region, the cell densities of *G. toxicus* were variable and affected by spatial and temporal variations. In Tanzania, it ranged from 0.0 to 879.5 (Bawe station) cells g^−1^ wet weight (ww) algae and 0 to 92.6 cells g^−1^ ww seagrass (Mbudya) [66]. The abundances on macroalgae in the Mayotte coral reef complex were reported to range from 0.0 to 2800 cells g^−1^ [60]. Monitoring of *G. toxicus* in the locality of Saint Leu (Reunion) since 1993 revealed high variability in population density with an average value of 122 ± 24 cells g^−1^ of algal turf [64]. In the coral reef complex of Mayotte Island (SW Indian ocean), the concentrations of *G. toxicus* ranged from 800–5400 dinoflagellates g^−1^ of biodeposits in the northeast lagoon. Up to 6000 dinoflagellates g^−1^ were recorded at Bambo islet in the southeast lagoon. Lower abundances up to 400 g^−1^ were recorded near the main island shore; seawards on the outer side of the barrier reef; and in luxurious coral areas more exposed to humans. At stations near the main island coast, abundance of *G. toxicus* was less than 100 cells g^−1^ [60]. The density of *G. toxicus* was as low as 0–4 cells g^−1^ of macrolagae in the lagoons of Trou Aux Biches, Mauritius [84].

In the northern part of the Indian ocean, the cell numbers of *Gambierdiscus* from the central Red Sea, Saudi Arabian coast were <40 cells g^−1^ ww of algae [43,44]. Similarly, *F. yasumotoi* in Kuwaiti shores had an average cell density of 116.7 ± 47.5 cells g^−1^ of ww algae. The high biomass algae (HABs) were estimated through the Ocean Color Modis Algorithm (OC3M), Garver-Siegel-Maritorena Algorithm (GSM), Generalized Inherent Optical Property (GIOP) model [41,45]; these areas when sampled showed the presence of *G. toxicus* in a concentration of ~1000 cells per liter of seawater [41].

### 3.3. Ciguatera Causative Organisms’ Substrates and Co-Occurring Species

*Gambierdiscus* is an epiphytic benthic dinoflagellate, commonly found on algal turfs, coral rubble, and macroalgae. A study from the Coastal region of Tanzania reported the *G. toxicus* strains along with unknown brown algae mixed with filamentous cyanobacteria species and the red alga *Gracillaris* sps. [66]. The dinoflagellate was also separated from two species of seagrass namely *Thalassia hemprichii* and *Thalasodendron ciliatum* in the same study [66]. The dinoflagellate assemblage observed in coral reefs revealed toxic species including *G. toxicus*, *Prorocentrum* spp., and *Ostreopsis* spp [85]. The co-occurrence of *Prorocentrum* sps. and *Ostreospis* sps. with *G. toxicus* were reported by Grzebyk and his group. In the same study, it was also demonstrated that some microalga stimulated whereas the others subsided the growth of *G. toxicus* [60]. Benthic thecate dinoflagellates in the sandy ecosystem of La Possession bay (Reunion Islands) were isolated as a complex of five toxic species namely *G. yasumotoi*, *G. toxicus*, *P. arenarium*, *P. concavum,* and *P. lima*. At St. Leu and Saline reef, 25 benthic thecate dinoflagellate species coexisted out of which 15 were harmful and are presented in Table 1 [65]. The benthic dinoflagellate of *G. toxicus* was reported to coexist with *Ostreopsis* spp., *Prorocentrum* spp., *Coolia monotis,* and *Amphidinium* sp. in the lagoon of Trou Aux Biches, Mauritius [84]. In Mayotte islands, South west of Indian Ocean, *G. toxicus* were reported along with *Prorocentrum* spp., *Ostreopsis* sp., and *Amphidinium* spp. [50,59].

In the reports of Catania and her team [43,44], the Red Sea strains of *G. belizeanus* were associated with the macroalgae species of *Turbinaria decurrens* and *Halimeda* sps. Information on macroalgal species associated with *G. belizeanus* isolated from the Gulf of Aqaba is unavailable [42]. In the case of *G. yasumotoi*, the macroalgal substrates were identified as *Padina tetrastomatica* and *Sargassum oligocystum* in winter (November 2012) collections whereas in summers (March 2013) they occurred along with *Chaetomorpha* sp. The group also reported the co-occurrence of macroalgal species of *Amphidinium carterae*, *Coolia monotis*, *Ostreopsis* sps., *Prorocentrum formosum*, *P. tsawwassenense*, *Peridinium quinquecorne*, *Adenoides eludens,* and *Cabra matta* [42]. Moderate Resolution Imaging Spectroradiometer (MODIS) and Medium Resolution Imaging Spectrometer (MERIS) data and its various operational algorithms such as OC3M-547, GSM, GIOP, and other bio-optical methods developed by Uddin and co-workers [41,45,54], revealed algal blooms; during field verification, it was observed that *G. toxicus* was also part of the algal bloom on 21 December 2009. Samples collected from location 29.38694° N, 47.78389° E had over 1000 cells/L. Other algae identified in the field samples were *G. toxicus*, *Karenia selliformii*, *K. brevis*, *P. lima*, *Dinophysis rotundata*, *Ceratium tripos*, and *Myrionecta rubrum*.

Recent deliberations by the experts in an international meeting in Rome on ciguatera poisoning recommended the next generation sequencing (NGS) to mass-amplify specific gene sequences from sediment samples to characterize all species or specific taxa present in the sample [8]. This approach allows better identification and resolution of microbial community composition than the conventional morphological and molecular methodologies. The information on ciguatera from the Indian Ocean region is still in its initial phase. This approach would provide comprehensive information on ciguatera diversity and its interaction with associated communities as reported elsewhere [87,88,89,90,91].

### 3.4. Ciguatera Causative Organism’s Morphology and Phylogeny

A variety of techniques are known to be used for ciguatera identification at a particular site. These include light microscopy (LM), scanning electron microscopy (SEM), DNA sequencing, restriction fragment length polymorphism (RFLP), quantitative polymerase chain reaction (qPCR), and metabarcoding [8,87]. While performing the morphological identification, the taxonomic key reported earlier [25] was followed by the research groups to confirm the species of *Gambierdiscus* found in the region. Earlier in the 1990s, SEM was used for the identification of *G. toxicus* from Reunion Islands [64]. SEM was also used recently to identify the species of *G. toxicus*; *G. belizeanus*; *G. polynesiensis*; *G. australes;* and *F. yasumotoi* [69]. The morphological identification of Gulf strains of *G. yasumotoi and G. belizeanus* was based on both LM and SEM [42,43,44]. A description of morphological parameters of strains present in the Indian Ocean region is given in Table 2.

Owing to the plasticity of morphological characteristics of some species of *Gambierdiscus,* the identification can be ambiguous if only morphology is used. Currently, molecular techniques are gaining popularity for the confirmation of species identified through microscopic methods. The only report on molecular identification of *Gambierdiscus* in the Arabian Gulf was by Catania [43] and her team [44]. The group amplified an 850 bp hypervariable (D8-D10) region of the larger subunit (LSU) of rRNA using primers FD8 and RB [11,25] through Sanger sequencing. A query of assembled nucleotides on the National Centre for Biotechnology and Informatics (NCBI) through the basic local alignment search tool (BLAST) resulted in a 100% match with *G. belizeanus*. Their (KY782637–KY782645) strains depicted a close resemblance with the Caribbean strain of *G. belizeanus*. The group also reported a 116 bp deletion at 493–609 position in one of the isolates, probably imparting the distinctiveness from other species of *Gambierdiscus* [95,96].

**Figure 8:**
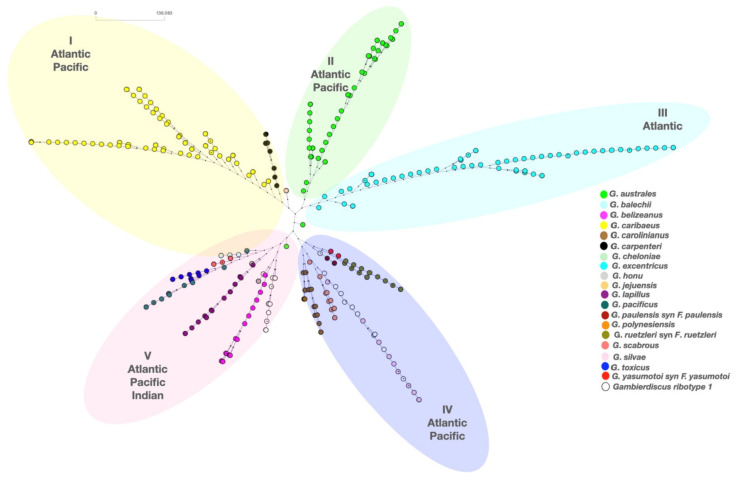
Phylogenetic associations of globally distributed *Gambierdiscus* and *Fukuyoa* species. A split decomposition algorithm was applied on multiple aligned D8-D10 sequences of LSU of available *Gambierdiscus* species (downloaded from NCBI) and aligned through ClustalW2.

Phylogenetic analysis (methodology described in the Supplementary file) based on multiple alignments (Clustal2.1) of all the available DNA sequences (n = 345) of the D8–D10 region of the large sub unit (LSU) of ribosomal RNA (rRNA) [9,11,14,15,25,26,40,43,44,89,96,97,98,99,100,101,102,103,104,105,106,107,108,109,110,111,112,113,114] distributes the *Gambierdiscus* genus into five major clades (clade I–V) as seen in Figure 8. Clade II and III exclusively contain the species *G. australes* (Atlantic and Pacific strains) and *G. excentricus* (Atlantic strains), respectively. Clade I encompass all of the *G. carribaeus* (Atlantic and Pacific strains) and newly discovered *G. carpenteri* (Atlantic and Pacific strains) as well as *G. jejuensis* (Pacific strains). Clade IV and V are more diverse. Of this, *G. belizeanus* (Red Sea, Saudi Arabia; Charco Azul, El Hierro, Spain; St. Barthelemy Island, Caribbean Sea) appears as a distinct branch in Clade V with the closest genetic relationship with *G. honu* (Kermadec Islands, Australia). The yet unclassified *Gambierdiscus* ribotype 1 follows the same lineage as of *G. belizeanus*. Other species in close proximity were *G. balechii* (Celebes Sea, Pacific Ocean; Phuket Islands, Indonesia, North Pacific), *G. lapillus* (Great Barrier Reef, Australia, Pacific Ocean; Cook Islands, Rarotonga, North Atlantic), *G. cheloniae* (Rarotonga, Cook Islands, North Atlantic), *G. scabrous* (Japan, South China Sea, North Pacific), and *G. pacificus* (Balearic Islands, Spain, North Atlantic; Cook Islands, Rarotonga, North Atlantic; Marshall Islands, Micronesia, South Pacific). The *G. toxicus* (Indian Ocean) strains are also in the same clade.

Molecular identification of *F. yasumotoi* on the Kuwaiti shore waters and Gulf of Aqaba have not yet been performed. Similarly, *G. australes*, *G. toxicus,* and *G. polynesiensis* from Manora Channel, Indian Ocean region are pending molecular identification. *F. yasumotoi* isolated from the Pacific [11] and Atlantic region fall in the same branch as that of *F. reutzleri* (North Atlantic and North Pacific Ocean) and *F. paulensis* (North Atlantic and South Pacific Oceans). All the *F. yasumotoi* strains discovered so far showed a similarity of ~80% with the unique ribotype A213 [11,97]. Other species in the same clade are that of *G. carolinianus* (Bermuda, North Atlantic; Bahamas, North Atlantic; Cancun, Mexico, North Atlantic; Caribbean Sea, North Atlantic), *G. silvae* (Canary Island, North Atlantic), *G. polynesiensis* (Cook Islands, Rarotonga, North Atlantic; Pacific Ocean), and unclassified *Gambierdiscus* ribotypes (Curlew Cay, Belize, North Atlantic).

More diversity in clade IV and V including the species discovered from Indian Ocean may be due to a smaller number of samples owing to the recent discovery of these species. The presence of unclassified *Fukuyoa* sps. and *Gambierdiscus* ribotypes in these clades is suggestive of unexplored novel species. In 2016, at the 32nd session of the Codex Committee on Fisheries and Fishery Products, the Pacific Nations raised CP as an issue that is increasingly affecting the tropical and subtropical regions of the Pacific Ocean, Indian Ocean and the Caribbean Sea between the latitudes of 35° N and 35° S [8]. The strains found in the Indian Ocean and their adjoining seas lie near the strains commonly found in these high-risk regions. Probably, the interconnection between the water bodies and the ballast water is the major source of ciguatera infiltration in the Indian Ocean waters. Given this, extensive monitoring and risk management program should be developed for the unexplored regions and specifically in the Middle East region.

### 3.5. Vectors of Ciguatera in Indian Ocean Region

The CP is recognized as a tropical disease but the existence of ciguatoxic fishes is reported globally due to international seafood trade and shipment [115]. According to a recent FAO/WHO report, globally 425 species of fish, especially those inhabiting the coral reefs, have been associated with CP [8]. The most significant toxic fish have been made by barracuda (Sphyraenidae), amberjack (*Seriola*), grouper (Serranidae), snapper (Lutjanidae), po’ou (*Labridae* spp.), jack (*Carangidae* spp.), trevally (*Caranx* spp.), wrasse (*Labridae* spp.), surgeonfish (*Acanthuridae* spp.), moray eel (*Muraenidae* spp.), roi (*Cephalopholis* spp.), parrotfish (*Scaridae* spp.), etc.

The CP cases have mainly been reported in the southwestern region encompassing Comoros, Mayotte, La Reunion, Mauritius, Rodrigues, and Seychelles. The reports from the Northern part of the Indian ocean have been scarce and involve cases in Mangalore and Trivandrum on the southern Indian coast and an outbreak resulting in one fatality from Pakistan, but the fish species involved and their origin could not be confirmed [74,75,116,117]. Few cases of CFP were also reported in Thailand after eating ocean fish particularly sea bass and red snapper [118,119]. One CP record from Egypt exists [120]. There has been no event recorded in the Northern part of the Arabian sea, in countries bordering the Gulf region or the Red Sea. The very first incidence of ciguatera poisoning in the Indian Ocean region was reported in 1993 from Manakara and Madagascar where about 500 persons were infected after consuming ciguatoxic shark. The fatality rate of this outbreak was 20% [121]. In Trivandrum, India, an autochthonous outbreak was reported in 2015 and 2016, due to which nearly 200 workers of a fish factory contracted CFP after eating heads of Red Snappers [75]. In 2013, also severe food poisoning events were witnessed after consuming a bull shark (*Carcharhinus leucas*), resulting in the deaths of 11 people in Madagascar in 2013 [78].

Fish originating from the Indian ocean region were implicated in ciguatera poisoning in other part of the world. The rapid alert system for food and feed (RASFF) created in 1979 by the European Union (EU) successively warned in 2012, 2015, and 2016 about ciguatoxic fishes originating from India and Sri Lanka (RASFF No. 2012-1602, 2015-0088, 2016-0932, 2016-1152, 2016-1155, 2017-1112). In 2016, scientists and regional public health authorities warned the population in Southwest India about the CFP risk caused by the consumption of Red Snappers [122], yet snappers from India were imported by France and distributed to Germany (RASFF No. 2016-0932). The French Poison Control center network reported from 2012 to 2019, 17 events with fish caught from the Indian Ocean. In a recent report from Germany, the rarely occurring CP outbreaks were reported between 2012–2017, the main reason identified as imports of snappers (Lutjanidae) from the Indian Ocean region mainly from countries like India, Indonesia, and Vietnam. The author emphasized that fishes from the Indian Ocean can cause ciguatera, which has been poorly documented [81].

**Figure 9:**
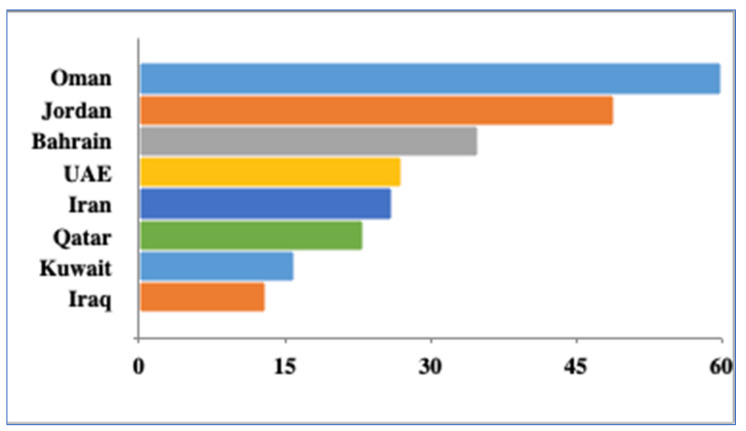
Number of Fishes associated with CP in Persian Gulf Countries as per the records of Fish Base (accessed on 1 June 2021).

Although incidences of CP have not been reported from the Arabian Gulf yet, however, the fishes such as grouper, snapper, emperors, barracuda, jacks, trevallies, kingfish, and tuna form the part of common catch by local fishermen in Arabian Gulf [123]. The database search in Fish base returned a minimum of 13 (Iraq) to a maximum of 60 (Oman) fishes associated with CP elsewhere in the world [124] (Figure 9). An increase in ciguatera causative organism could have a major impact on food safety and food security given that those species are highly consumed in the region. The suspicion of a high number of unreported cases is warranted given that ciguatera is difficult to diagnose and not subject to mandatory reporting [125].

The Persian Gulf water is the only source of freshwater through seawater desalination to most Gulf countries and an important source of seafood for the region. Previous reports on incidences of harmful algal blooms on drinking water and food safety and of massive fish kills in the region further creates awareness and interest to investigate the occurrence of ciguatera causative organisms and associated toxins, as well as their health implications in the Gulf countries [58].

### 3.6. Ciguatera Monitoring in Indian Ocean Region

Risk assessment and monitoring of Ciguatera in the Indian Ocean region is largely lacking with only a couple of previous attempts from la Reunion [47] and Mayotte Island [61]. In 2014, an algal bloom monitoring system was developed for Kuwait’s coastal waters. The OC3M (Aqua-MODIS) and OC4E (ENVISAT-MERIS) algorithms most accurately measured chlorophyll-a concentrations in Kuwait bay. Due to the poor temporal resolution and the decommissioning of ENVISAT-MERIS, Aqua-MODIS data was used for continuous observation. Additionally, algorithms such as Generalized Inherent Optical Properties (GIOP), Garver, Siegel, Maritorena Model, OC2, MODIS fluorescence line height, and the MERIS-based NIR-Red algorithms were attempted, which have a lower accuracy when compared to the OC3M algorithm. The OC3M detected the most reported in-situ algal bloom events (19/50) and most accurately measured chlorophyll-a concentration (RMS: 2.42, RMSE: 4.11, Mean Bias: 54.2%). The Aqua-MODIS OC3M was selected as the preferred algorithm to monitor chlorophyll-a concentration and to detect algal blooms in Kuwait bay and surrounding waters [41,45]. Data variables such as sea surface temperature, OC3M, distance to aquaculture, Garver-Siegel-Maritorena (GSM), generalized inherent optical properties (GIOP), euphotic depth, Secchi disk depth, distance to shore, precipitation, photosynthetically active radiation (PAR), distance to the river, bathymetry, colored dissolved organic matter, wind direction, speed, and precipitation, etc. were also estimated through a multivariate regression model, a hybrid multivariate regression model, an artificial neural network model, and a hybrid artificial neural network model by Uddin and his group [41,45].

A synergistic model was created that combines the GIS-imaging, the different estimates of environmental parameters, and in situ monitoring with traditional toxin analytical methods [41,54]. Sampling epiphytic substrates and analyzing samples using traditional optical microscopy will provide very useful and immediate information and developing ciguatera early warning systems in the region. The molecular methods of qPCR and metabarcoding can be useful but only as a complement not as the basic methodology to estimate *Gambierdiscus* abundance.

## 4. Conclusions

Ciguatera research in the Indian Ocean region is limited and fragmented. Although, there are reported cases of CP in the region. The most significant toxic fish in the region were barracuda (Sphyraenidae), amberjack (*Seriola*), grouper (Serranidae), snapper (Lutjanidae), po’ou (*Labridae* spp.), jack (*Carangidae* spp.), trevally (*Caranx* spp.), wrasse (*Labridae* spp.), surgeonfish (*Acanthuridae* spp.), moray eel (*Muraenidae* spp.), roi (*Cephalopholis* spp.), parrotfish (*Scaridae* spp.), seabass, shark, and red snapper to name a few. Since the region exports fisheries, some of the fish originating from India and Sri Lanka in 2012, 2015, and 2016 were implicated in ciguatera poisoning in Europe.

A comprehensive survey of algal substrates in the region complemented with high throughput metabarcoding would provide insights into novel and undiscovered contributors of ciguatera. Although less in numbers, the presence of ciguatera causative organisms in the region cannot be ignored and their interaction with substrate and other microbial species is worthy of further investigation. The development of an early warning system for HABs is very much the need of the hour.

## Figures and Tables

**Table 1:** Occurrence of *Gambierdiscus* species in the Indian Ocean region.

Species	Location	Marginal Seas	GPS Coordinates	Sampling Depth	Temperature	Associated Species	Salinities	Reference
*G. toxicus*	Kuwait	Persian Gulf	29.3860° N, 47.7830° E		17.59 °C	*Karenia selliformis*, *Karenia brevis*, *Prorocentrum lima*, *Phalacroma rotundatum*, *Tripos muelleri* and *Mesodinium rubrum*		[41,45]
*F. yasumotoi*	Qit’at Julai’ah, Kuwait	Persian Gulf	28.8370° N, 48.8160° E	1–3 m	21–22.3 °C	*Padina tetrastomatica*, *Sargassum oligocystum*, *Chaetomorpha* sp., *Amphidinium carterae*, *Coolia monotis*, *Ostreopsis* sps., *Prorocentrum formosum*, *P. tsawwassenense*, *Blixaea quinquecornis*, *Adenoides eludens* and *Cabra matta*	41.2–42.4	[42]
*F. yasumotoi*	Semi protected shallow lagoons, Kuwait	Persian Gulf	28.8530° N, 48.8230° E	1–3 m	23.5–25 °C		41.2–42.4	[42]
*F. yasumotoi*	Southern coast, Kuwait	Persian Gulf	28.8530° N, 48.8230° E	1–3 m	23.5–25 °C		42.1–42.8	[42]
*F. yasumotoi*	Jordan	Gulf of Aqaba	29.8250° N,34.8580° E	1.5–2 m	24–25 °C	NA	40.0	[42]
*G. belizeanus*	Jordan	Gulf of Aqaba	29.8250° N, 34.8580° E	1.5–2 m	24–25 °C	NA	40.0	[42]
*G. belizeanus*	Qita Al Kirsh, Saudi Arabia	Red Sea	22.6058° N, 39.1980° E	0.5–0.6 m	29.3 °C	*Turbinaria decurrens* and *Halimeda* sp.	38.4	[43,44]
*G. belizeanus*	Sh’ib Nazar, Saudi Arabia	Red Sea	22.4916° N, 38.9722° E	0.4 m	30.1 °C	*Turbinaria decurrens*	28.9	[43,44]
*G. belizeanus*	Malathu Reef, Saudi Arabia	Red Sea	19.8194° N, 39.9194° E	0.5–0.7 m	NA	*Turbinaria decurrens*	NA	[43,44]
*G. belizeanus*	Marmar Reef, Saudi Arabia	Red Sea	19.9038° N, 39.9947° E	0.8 m	NA	*Turbinaria decurrens*	NA	[43,44]
*G. belizeanus*	Al Fahal, Saudi Arabia	Red Sea	22.5386° N, 38.9813° E	0.6–0.8 m	30.4 °C	*Turbinaria decurrens* and *Halimeda* sp.	36.0	[43,44]
*G. belizeanus*	Abu Shosha, Saudi Arabia	Red Sea	22.3505° N, 39.2811° E	0.5 m	29.6 °C	*Turbinaria decurrens* and *Halimeda* sp.	38.3	[43,44]
*G. belizeanus*	Um Al Balam, Saudi Arabia	Red Sea	22.4277° N, 38.9652° E	0.4–0.6 m	29.3 °C	*Turbinaria decurrens* and *Halimeda* sp.	36.1	[43,44]
*G. belizeanus*	Um Al Kiethl, Saudi Arabia	Red Sea,	22.3197° N, 39.0541° E	0.5 m	29.5 °C	*Turbinaria decurrens* and *Halimeda* sp.	28.9	[43,44]
*G. belizeanus*	Coast Guard Reef, Saudi Arabia	Red Sea	20.3950° N, 40.3580° E	0.5 m	NA	*Turbinaria decurrens* and *Halimeda* sp.	NA	[43,44]
*G. belizeanus*	Mangrove Reef, Saudi Arabia	Red Sea	20.4400° N, 40.2911° E	0.4 m	NA	*Turbinaria decurrens*	NA	[43,44]
*G. toxicus*	Sindh Coast, Pakistan	Manora Channel; Chuma Island	24.8091° N, 66.9908° E	1.0 m	32 °C			[69]
*G. toxicus*	Balochistan Coast, Pakistan	Manora Channel; Chuma Island	24.7861° N, 66.4516° E	1.0 m	28 °C			[69]
*G. belizeanus*	Sindh Coast, Pakistan	Manora Channel; Chuma Island	24.8091° N, 66.9908° E	1.0 m	32 °C			[69]
*G. belizeanus*	Balochistan Coast	Manora Channel; Chuma Island, Pakistan	24.7861° N, 66.4516° E	1.0 m	28 °C			[69]
*G. australes*	Sindh Coast	Manora Channel; Chuma Island, Pakistan	24.8091° N, 66.9908° E	1.0 m	32 °C			[69]
*G. australes*	Balochistan Coast	Manora Channel; Chuma Island, Pakistan	24.7861° N, 66.4516° E	1.0 m	28 °C			[69]
*G. polynesiensis*	Sindh Coast	Manora Channel; Chuma Island, Pakistan	24.8091° N, 66.9908° E	1.0 m	32 °C			[69]
*G. polynesiensis*	Balochistan Coast	Manora Channel; Chuma Island, Pakistan	24.7861° N, 66.4516° E	1.0 m	28 °C			[69]
*F. yasumotoi*	Sindh Coast	Manora Channel; Chuma Island, Pakistan	24.8091° N, 66.9908° E	1.0 m	32 °C			[69]
*F. yasumotoi*	Balochistan Coast	Manora Channel; Chuma Island, Pakistan	24.7861° N, 66.4516° E	1.0 m	28 °C			[69]
*Gambierdiscus* sp.	Bay of Bengal	India						[71]
*G. toxicus*	Mbudya Island, Dar es Salaam	Tanzania		5.0 m	25.5–31.0 °C	Seagrass—*Thalassodendron ciliatum*	35–37	[66]
*G. toxicus*	Bawe Island, Zanzibar	Tanzania		10.0 m	26.0–29.0 °C	Unknown brown algae mixed with Filamentous cyanobacteria	34–36	[66]
*G. toxicus*	Makoba, Zanzibar	Tanzania		10.0 m	26.0 °C	*Gracilaria* sp.	35–43	[66]
*G. toxicus*	Longoni Bay (Stations 1 and 2)	Mayotte, Mozambique	12.8333° N, 45.1666° E	Between the depth of the lower intertidal level and 30 m	26–31 °C	*Ostreopsis lenticularis*; *O. lenticularis*; *Prorocentrum lima* and 4 new species of *Prorocentrum*	35–37	[60]
*G. toxicus*	Surprise Reef (Station 3)	Mayotte, Mozambique	12.8333° N, 45.1666° E					[60]
*G. toxicus*	Prevoyante Reef (Station 5)	Mayotte, Mozambique	12.8333° N, 45.1666° E					[60]
*G. toxicus*	Great Barrier Reef (Station 4A–C)	Mayotte, Mozambique	12.8333° N, 45.1666° E					[60]
*G. toxicus*	Cape Andrema Station 6)	Mayotte, Mozambique	12.8333° N, 45.1666° E					[60]
*G. toxicus*	M’Songoma (Station 7)	Mayotte, Mozambique	12.8333° N, 45.1666° E					[60]
*G. toxicus*	M’Sanga Tsohole Reef (Station 8A,B)	Mayotte, Mozambique	12.8333° N, 45.1666° E					[60]
*G. toxicus*	Bambo Reef (Station 9A,B)	Mayotte, Mozambique	12.8333° N, 45.1666° E					[60]
*G. toxicus*	Mayotte	South West Indian Ocean				*Prorocentrum* spp., *Ostreopsis* sp., *Amphidinium* spp		[59]
*G. toxicus*	Mayotte	South West Indian Ocean						[86]
*G. belizeanus*	St-Leu and Saline reef	Reunion Island	21.1533° N, 55.2944° E	1–5 m		*P. arenarium*; *P. belizeanum*; *P. concavum*; *P. elegans*; *P. emarginatum*; *P. hoffmannianum*; *P. lima*; *P. mexicanum*; *P sculptile*; *P. borbonicum*; *O. heptagona*; *O. labens*; *O. lenticularis*; *O. mascarensis*; *O. ovata*; *O. siamensis*; *C. monotis*; *C. tropicalis*; *Sinophysis microcephalis*; *S. canaliculate*; *Amphidiniopsis* sp.; *Bysmatrum* sps.		[65]
*G. belizeanus*	Possession Bay	La Reunion	20.9347° N, 55.3491° E	10–12 m		*P. arenarium*; *P. emarginatum*; *P. concavum*; *P. lima*; *P panamensis*; *Coolia* sps.		[65]
*G. toxicus*	St-Leu and Saline reef	La Reunion	21.1533° N, 55.2944° E	1–5 m		*P. arenarium*; *P. belizeanum*; *P. concavum*; *P. elegans*; *P. emarginatum*; *P. hoffmannianum*; *P. lima*; *P. mexicanum*; *P sculptile*; *P. borbonicum*; *O. heptagona*; *O. labens*; *O. lenticularis*; *O. mascarensis*; *O. ovata*; *O. siamensis*; *C. monotis*; *C. tropicalis*; *Sinophysis microcephalis*; *S. canaliculate*; *Amphidiniopsis* sp.; *Bysmatrum* sps.		[65]
*G. toxicus*	Possession Bay	La Reunion	20.9347° N, 55.3491° E	10–12 m		*P. arenarium*; *P. emarginatum*; *P. concavum*; *P. lima*; *P panamensis*; *Coolia* sp.		[65]
*F. yasumotoi*	St-Leu and Saline reef	La Reunion	21.1533° N, 55.2944° E	1–5 m		*P. arenarium*; *P. belizeanum*; *P. concavum*; *P. elegans*; *P. emarginatum*; *P. hoffmannianum*; *P. lima*; *P. mexicanum*; *P sculptile*; *P. borbonicum*; *O. heptagona*; *O. labens*; *O. lenticularis*; *O. mascarensis*; *O. ovata*; *O. siamensis*; *C. monotis*; *C. tropicalis*; *Sinophysis microcephalis*; *S. canaliculate*; *Amphidiniopsis* sp.; *Bysmatrum* sp.		[65]
*F. yasumotoi*	Possession Bay	La Reunioin	20.9347° N, 55.3491° E	10–12 m		*P. arenarium*; *P. emarginatum*; *P. concavum*; *P. lima*; *P panamensis*; *Coolia* sp.		[65]
*G. toxicus*	Reunioin Islands	Mauritius				*Prorocentrum* sp.; *Ostreopsis* sp.		[64]
*Gambierdiscus*	Mangalore	India	12.91777° N, 74.79162° E					[72,73,74]

NA-Not available.

**Table 2:** Morphological features of *Gambierdiscus* species found in the Indian Ocean region.

	*F. yasumotoi* [12]	*G. belizeanus* [92]	*G. toxicus* [93]	*G. polynesiensis* [94]	*G. australes* [94]
Cell depth	54 to 73 µm	59 to 73 µm	71 to 96 µm	65 to 87 µm	60 to 82 µm
Cell width	46 to 61 µm	60 to 73 µm	45 to 140 µm	64 to 75 µm	65 to 85 µm
Cell length	49 to 70 µm	47 to 51 µm	50 to 150 µm	57 to 75 µm	76 to 93 µm
Cell shape	Globular	Round	Rounded/ellipsoid	Rounded/ellipsoid	Rounded/ellipsoid
Apical pore	Fish hook-shaped	Fish hook-shaped	Fish hook shaped	Fish hook shaped	Fish hook shaped
Thecal surface		Moderately aerated		Smooth	Smooth
Apical plate	elongated and teardrop-shaped	Ellipsoid	Ellipsoid	large triangular apical pore plate	broad ellipsoid apical pore plate
Hypotheca		Truncate-conical			Eight hypothecal plates
Epitheca		dome-shaped	Ellipsoid		Eleven plates
Sulcus			Short		
Chloroplast	elongated golden-brown chloroplasts were peripherally arranged	golden-brown drop-shaped chloroplasts radiating from the cell centre			
Nucleus	large, oblong and located in the right posterior	large, oblong and crescent-shaped, and located in the right posterior cell part			Posteriorly situated nucleus
Cingulum		deeply excavated, descending,	Ascending narrow cingulum		Narrow and deep
Plate Formulae			Po, 3′, 7″, 6C, 8S, 5‴, 1p, 2⁗	Po, 3′, 7″, 6C, 8S, 5‴, 1p et 2‴	Po, 3′, 7″, 6C, 8S, 5‴, 1p et 2⁗
Studied by	[42,69]	[42,43,44,69]	[66,69]	[69]	[69]

## Data Availability

Data provided as Supplementary file and within manuscript itself.

## Supplementary Materials

The following are available online at https://www.mdpi.com/article/10.3390/toxins13080525/s1, Metadata and percent identity matrix of 345 Gambierdiscus sequences downloaded from NCBI and aligned using Clustal W2.1.
